# Homoclinic and Heteroclinic Orbits in Climbing Cucumber Tendrils

**DOI:** 10.1038/s41598-019-41487-5

**Published:** 2019-03-25

**Authors:** Jingjing Feng, Wei Zhang, Cheng Liu, Ming Guo, Chunqiu Zhang

**Affiliations:** 1 Tianjin Key Laboratory of the Design and Intelligent Control of the Advanced Mechatronic System, School of Mechanical Engineering, Tianjin University of Technology, Tianjin, 300384 China; 2 Beijing Key Laboratory on Nonlinear Vibrations and Strength of Mechanical Structures, Beijing University of Technology, Beijing, 100124 China; 3 National Demonstration Center for Experimental Mechanical and Electrical Engineering Education, Tianjin University of Technology, Tianjin, 300384 China; 4 Tianjin KunLun Decoration Engineering Company, Tianjin, 300191 China

## Abstract

Many biomaterials utilize chiral growth to imitate biological functions. A prominent example can be found in growing cucumbers, which use tendrils as winding support for both fixation and climbing. A number of tendril-mimicking materials and artificial plant-like mechanical machines have been developed to imitate tendril deformation. However, tendrils tend to not only show spiral or parallel shapes, but also a combination of both configurations. It remains unclear whether these morphologies are regular and how they form mechanically. Here, the morphology of climbing tendrils as a complex nonlinear phenomenon is investigated via experimental and theoretical approaches. The results of the experiments clarify the relationship between tendril morphologies and actual tendril growth as well as relevant stress characteristics during the climbing of a support by the tendril, and their mechanical properties. On this basis, the three-dimensional configuration problem of a cylinder-constrained rod has been utilized to describe the phenomenon of a tendril climbing support. The phenomena of spiral and parallel configuration combinations in tendrils could be effectively explained by studying similar homoclinic and heteroclinic orbits. Applying these results accurately guides the development of mimicking material.

## Introduction

Many biological materials use a variety of three-dimensional shapes in their growth^1–3^ and tendrils are a prominent example. Chiral growth plays a significant role in the biological function of biological materials. Growing cucumbers bind and climb by twining tendrils around a supporting structure and these cucumber tendril coils and overwinds can be applied to mechanics and biology^4–11^. The renowned contortion phenomenon of tendrils, which can also be found in fibres and other materials, has been investigated from many perspectives^12–16^. The obtained principles have been effectively applied to a variety of fields such as artificial plant-like mechanism design^17,18^, plant mimicking material fabrication^19–21^, and growth regulation mechanisms^22–24^.

**Figure 1:**
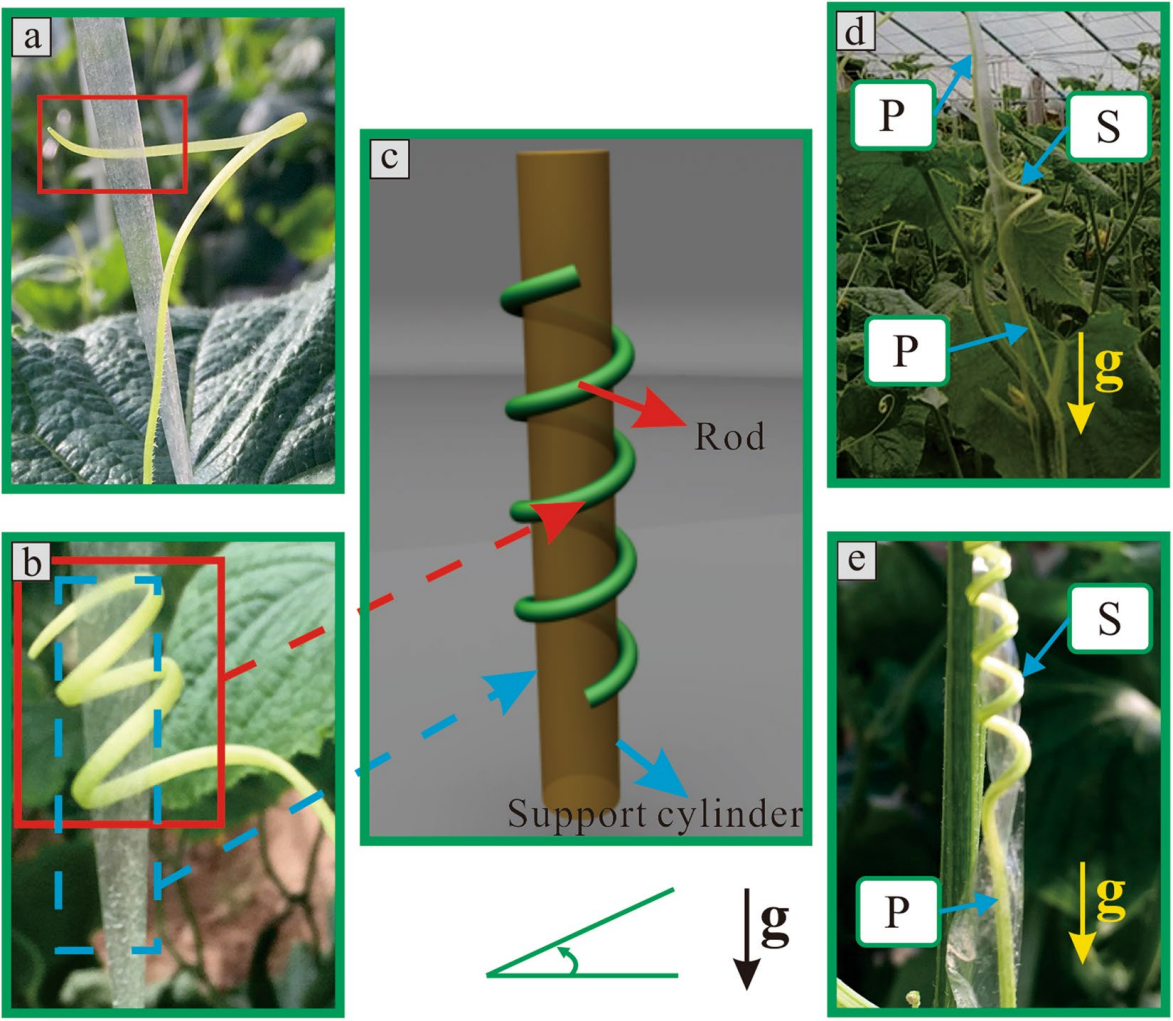
Tendrils climbing on support structures. (**a**) First contact with the support structure to climb up. (**b**) Climbing up along the support. (**c**) Physical model. (**d**,**e**) Specific configurations of tendrils climbing on supports. “g” represents the direction of gravity. “S” represents the spiral state of the tendril. “P” represents the parallel state of the tendril along the direction of its support.

In nature, chiral growth exists widely in biological materials. As shown in Fig. 1(a), once tendrils touch their support, they will either twist up or down along their support. Furthermore, they deform so that they can hold onto their support, as shown in Fig. 1(b). Therefore, spiral growth, which is represented by the letter “**S**” in Fig. 1(d,e), is the most common configuration of tendrils that are climbing on a support. Furthermore, parallel growth along the support enables tendrils to achieve maximum climbing height with minimal consumption, with the goal to obtain valuable resources such as sunlight. Therefore, a parallel state with a straight shape along the direction of supporting structures, which is represented by the letter “**P**” in Fig. 1(d,e), can also be found in the tendril configuration. Moreover, a combination of chiral and parallel configuration can also be found in tendrils, as shown in Fig. 1(d,e); however, the mechanistic mechanism of these tendrils has not been studied to date.

In addition to biological materials, many other engineering problems can be modelled as an elastic thin rod constrained by a cylindrical surface^25–33^. Research on the mechanics of a constrained rod achieved a comprehensive and profound breakthrough^34–43^, although the Kirchhoff theory is inapplicable due to the force caused by surface contact. Existing equations with different variables can be utilized to describe the three-dimensional configuration of a rod constrained by a cylinder. However, these equations contain complex nonlinear terms, which significantly complicate the research. Therefore, most studies only address the equilibrium problem of solutions in these equations^40,42–45^. Almost all of these equations have homoclinic and heteroclinic orbits (HAHOs); however, only few scholars utilize numerical simulations to draw phase diagrams of the systems^42–45^. The effects of HAHOs in these equations for the subsequent physical analysis have not been thoroughly investigated. To study the configuration of rods, the shooting method has been applied to investigate the approximate variation tendency demonstration of the rod^42–45^. From an analytical perspective, a qualitative summary of the characteristics of the specific shape of the rod has not been included.

In a previous study^46^, heteroclinic orbits were utilized as part of the explanation of a tendril winding knot. However, in the reported study only one set of data was utilized to draw the configuration of the rod; however, that data did not involve an actual force analysis or physical parameters. Therefore, the configurations of climbing tendrils under actual parameters and their formation mechanism remained unknown. Further questions that merit investigation focus on the physical parameters related to the previously described know phenomenon^46^ and the prediction of other configurations of climbing tendrils. These relevant and unresolved problems motivated this study. Here, the qualitative relationship between the morphology of climbing tendrils and the complex nonlinear phenomenon is studied via experimental and theoretical approaches.

## Plant Morphology and Mechanical Properties

### Growth Experiments

The growth processes of tendrils in their natural state were recorded. The deformations of cucumber tendrils are sufficiently slow to be regarded as a quasi-static process. A growth process video of a cucumber tendril climbing a support is shown as Supplemental material 1, which is a 1024-fold accelerated version. Due to this slow deformation, in mechanics, the growth process of a single tendril climbing a support can be regarded as a configuration problem of a rod that is constrained by a cylinder, as shown in Fig. 1(c).

According to the morphological characteristics of tendril growth, the following hypotheses can be established to analyse both the force and movement during the deformation process. The tendril gradually contacts its support. The process of its touching of a support from a dynamic to a static state is primarily discussed. The forces that act on it can be combined as a resultant growth force, which acts on its centre line. This centre line is only subject to bending and twisting deformation, without elongation or shortening. The entire growth process maintains constant speed from the initial tendril contact with its support to its morphological changes. The friction and collision that may occur when it touches support have been ignored, due to the slowness of deformation during this growth process.

**Figure 2:**
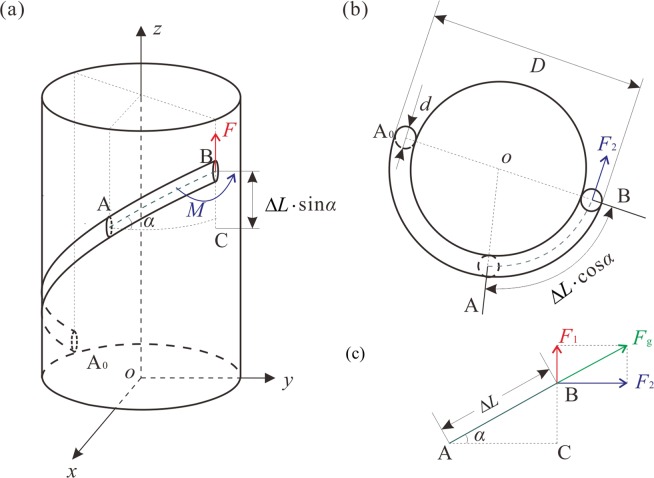
The growth model of tendril deformation during the process when a tendril climbs a support structure. (**a**) Three-dimensional graphic. (**b**) Top view. (**c**) Centre line of segment AB.

Moreover, the growth model of tendril deformation when the tendril climbs a support is established to measure the effective physical parameters, as shown in Fig. 2. Figure 2(a) shows when a tendril touches a support from the initial point A_0_, and then deforms to point B after a period of time. Point C is within this deformation process. The time and distance form point C to point B are Δ*t* and Δ*L*, respectively. This process satisfies Newton’s second law:

1 $${\boldsymbol{F}}=\frac{{\rm{d}}({\boldsymbol{P}})}{dt},$$

where ***F*** represents the force and ***P*** represents the momentum. The momentum is the product of mass *m* and velocity ***v***:

2 $${\boldsymbol{P}}={m}{\boldsymbol{v}}.$$

The winding deformation rate of a tendril remains constant; therefore, the velocity is independent of time. Furthermore, the value of ***v*** can be represented by the average velocity over a period of time:

3 $$v=\frac{{\rm{\Delta }}L}{{\rm{\Delta }}t}.$$

Considering that the mass varies uniformly over time, *m* is related to time, and the following transformation can be performed:

4 $$\frac{{\rm{d}}m}{{\rm{d}}t}=\frac{{\rm{\Delta }}m}{{\rm{\Delta }}t}.$$

Therefore, Eqs (2) and (4) are substituted into Eq. (1), and Eq. (5) can be obtained:

5 $${\boldsymbol{F}}=\frac{{\rm{\Delta }}m}{{\rm{\Delta }}t}{\boldsymbol{v}}.$$

At point B, the ***P*** follows the direction of tendril growth and the applied ***F*** is the growth force *F*_*g*_. The angle between segment AB and the horizontal line is *α* in Fig. 2(c). ***v*** can be decomposed into both the horizontal and vertical directions, which are written as follows:

6 $${v}_{{\rm{h}}}=\frac{{\rm{\Delta }}L}{{\rm{\Delta }}t}\,\cos \,{\alpha },$$

7 $${v}_{{\rm{v}}}=\frac{{\rm{\Delta }}L}{{\rm{\Delta }}t}\,\sin \,{\alpha },$$

Combined with Fig. 2(c), ***F***_*g*_ can be decomposed into the components ***F***_1_ and ***F***_2_:

8 $${{\boldsymbol{F}}}_{{\rm{g}}}={{\boldsymbol{F}}}_{1}+{{\boldsymbol{F}}}_{2}.$$

By substituting Eqs (6), (7), and (8) into Eq. (5), the forces ***F***_1_ and ***F***_2_ can be written as follows:

9 $${F}_{1}=\frac{{\rm{\Delta }}m}{{\rm{\Delta }}t}(\frac{{\rm{\Delta }}L}{{\rm{\Delta }}t})\sin \,{\alpha },$$

10 $${F}_{2}=\frac{{\rm{\Delta }}m}{{\rm{\Delta }}t}(\frac{{\rm{\Delta }}L}{{\rm{\Delta }}t})\cos \,{\alpha }.$$

As shown in Fig. 2(a), force ***F***_1_ can be regarded as causing segment AB to move upward, which can be rewritten as ***F***. The force ***F***_2_ can cause a bending movement of segment AB around the support *o*, which is shown as Fig. 2(a,b). The resulting force moment can be calculated by the following relation:

11 $$M={F}_{2}(\frac{D-d}{2})$$

The length density of segment AB, which is denoted as *ρ*, can be calculated by dividing its mass Δ*m* by its length Δ*L*:

12 $$\rho =\frac{{\rm{\Delta }}m}{{\rm{\Delta }}L}.$$

Therefore, by combining Eqs (9), (10), (11), and (12), the average force *F* and the force moment *M* acting on the tendrils and deforming the tendrils winding upwards along the support can be written as follows:

13 $$F=\rho {(\frac{{\rm{\Delta }}L}{{\rm{\Delta }}t})}^{2}\,\sin \,{\alpha },$$

14 $$M=\rho (\frac{D-d}{2}){(\frac{{\rm{\Delta }}L}{{\rm{\Delta }}t})}^{2}\,\cos \,{\alpha }.$$

According to the physical parameters presented in Eqs (13) and (14), a series of physical data of the climbing tendril can be recorded. The tendril samples are selected for observation without any external interference. Physical data related to sample growth are measured at regular intervals. Experimental data was measured when the actual growing tendrils finally showed the knot phenomenon, and the results are listed in Table 1.

**Table 1:** Experimental data of effective physical parameters as tendrils winding a knot.

	Δ*L* (mm)	Δ*t* (s)	*D* (mm)	*d* (mm)	*α* (°)	*l* (mm)	*m* (×10^−6^ kg)
1	6.74	6840	3.58	1.33	15	5.67	7.2
2	3.0	5760	3.58	1.33	15	1.89	2.4
3	9.38	4080	7.04	1.07	22	9.38	7.9
4	7.38	8100	7.26	1.15	36	7.38	6.8
5	2.49	3540	3.24	1.12	17.9	2.49	3.0
6	3.69	3660	3.24	1.12	17.9	2.69	3.8
7	1.42	2000	3.24	1.12	17.9	1.42	1.8
8	3.21	3780	3.24	1.12	17.9	3.21	3.2
9	2.3	740	3.24	1.12	17.9	2.3	1.5
10	4.0	1740	3.24	1.12	17.9	4.0	3.8
11	1.26	6720	5.28	1.21	3.73	7.43	8.2
12	15.79	10020	4.89	1.25	20.1	15.79	18.5
13	7.82	10840	4.89	1.25	20.1	7.82	13.3
14	8.93	4260	4.89	1.25	20.1	8.93	18.0
15	2.46	3260	6.13	1.25	20.05	5.56	4.2

During the process of growth, the average growth rate can be calculated by collecting both the length Δ*L* and the growing time Δ*t* between any two points A and B on a tendril climbing a support. After deformation, i.e., when the tendril presents a knot with a stable bending shape, other data of this collected sample can be measured. The diameter *d* and the outer diameter *D* of the entire deformed tendril wounding around the support are obtained, and the average inclination angle *α* can be calculated as the angle between the centre line of tendril and the support, as shown in Fig. 2(b). The length density *ρ* can be calculated with the length *l* and weight *m* between any two points on this sample.

### Tensile Testing

The mechanical properties of any biomaterial, such as a tendril, are studied because the mechanical properties of materials are very important for their mechanical analysis. In tensile testing, the mature tendrils of cucumbers were collected as samples from the same growing environment and site where the last experiments have been completed. The tendril samples are kept for 24 h at room temperature after their harvest.

**Figure 3:**
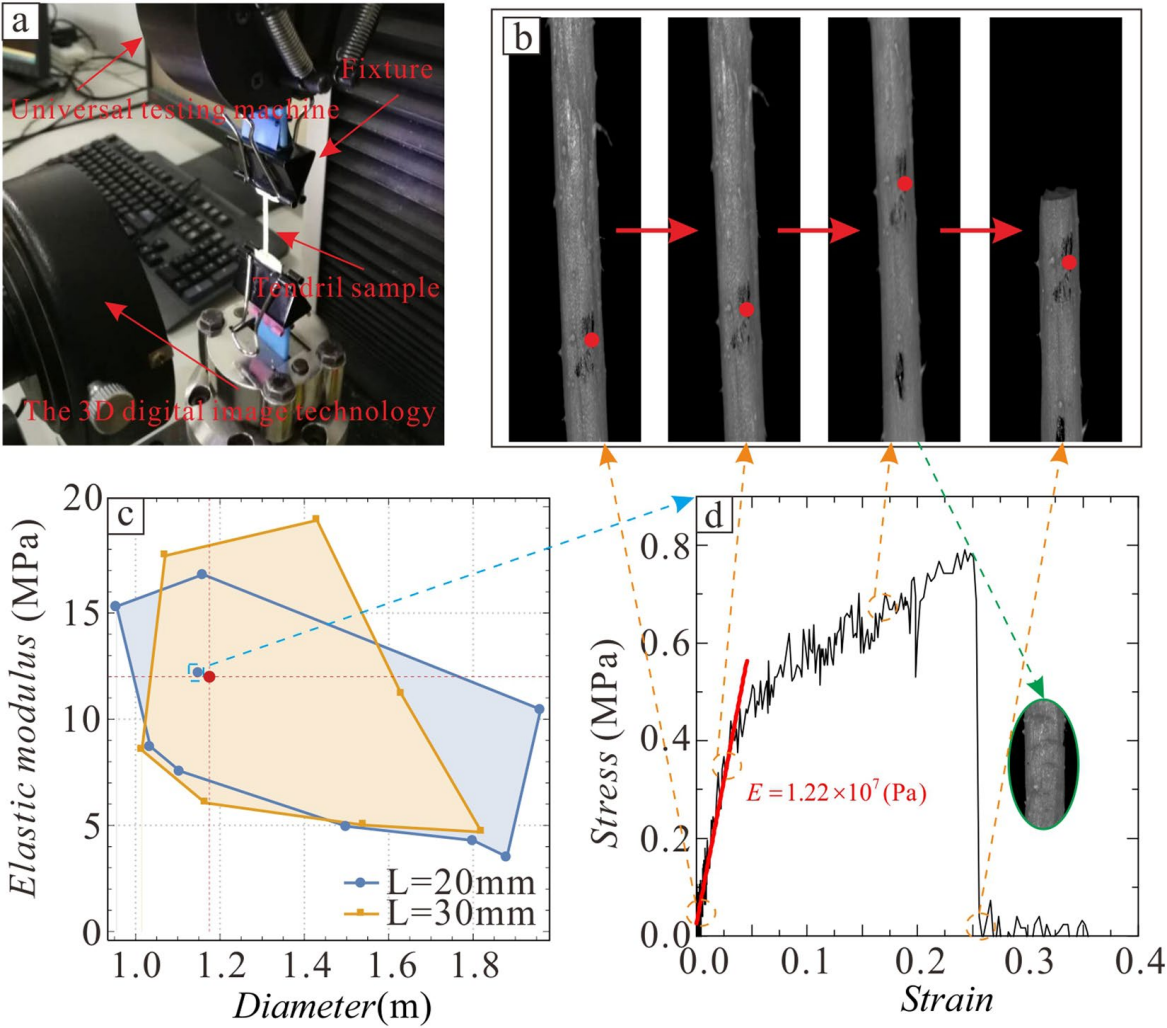
Tensile test of a tendril. (**a**) Physical drawings of tensile testing. (**b**) Several representative states in the strain-stress curve are the initial state, the elastic deformation state, the plastic deformation state, and the breaking state. (**c**) Large probability data range area of diameter-elastic modulus. (**d**) Strain-stress curve of a tendril in tension and the tearing phenomenon.

Tendrils are a uniform material and the samples are divided into two groups according to different effective lengths (L) of 20 mm and 30 mm. Then, 10 samples with different diameters (*d*) were studied in each group. As shown in Fig. 3(a), the sample was fixed to the universal testing machine via a self-made fixture, which was used to prevent tendril damage by standard fixtures. Three-dimensional (3D) digital image technology was used to collect relative displacement data.

In the stretch process, the tendril first showed elastic deformation, followed by plastic deformation, and the tendril surface was torn and finally broke during plastic deformation, as shown in Fig. 3(b). The tensile properties of both tendrils and plastic materials are basically similar. The cross-section shrinkage of tendrils is approximately between 9% and 15%, and the elongation is approximately between 7% and 15%. The changing rule of the tendril strain-stress curve is qualitatively similar under different diameters; therefore, one group of samples was used as example and is shown in Fig. 3(d).

The slope of the approximate linear part of the strain-stress curve is calculated via fitting, resulting in the tendril elastic modulus *E*. The tensile test data for all 20 samples are listed in Table 2. The large probability data range areas of the diameter-elastic modulus can be plotted by connecting the obtained (*d*, *E*) data points and retaining only those data points on the boundary of this area, as shown in Fig. 3(c). The ranges of both groups basically overlap under corresponding L. Therefore, it can be considered that *E* corresponding to *d* in this range should appear in these overlapping areas. The red point in Fig. 3(c) indicates the average diameter in this region. Then, the approximate value of the average elastic modulus can be obtained as *E* = 1.2 × 10^7^ Pa, which was used for the subsequent analysis. Figure 3(d) shows sample data near this approximate average value.

**Table 2:** Tensile test data of the Diameter-Elastic modulus under different effective lengths.

	L = 20 mm		L = 30 mm	
	*d* (mm)	*E* (GPa)	*d* (mm)	*E* (GPa)
1	1.960	10.46	1.820	4.68
2	1.800	4.29	1.430	19.37
3	1.880	3.52	1.630	11.18
4	1.160	16.82	1.540	5.01
5	1.500	4.95	1.165	6.06
6	1.035	8.72	1.015	8.54
7	1.105	7.56	1.070	1.77
8	0.955	15.31	1.170	9.93
9	1.150	12.20	1.165	14.78
10	1.115	13.5	1.130	15.2

### Morphological Experiments

For the qualitative observation of tendril morphology, tendrils that have formed a stable curved shape under natural growth are observed. In previous experiments, further combination configurations were found in tendrils; therefore, morphological experiments were conducted to record these particular configurations to summarize their morphological laws. Combinations of chirality and parallel state configurations of climbing tendrils were recorded.

**Figure 4:**
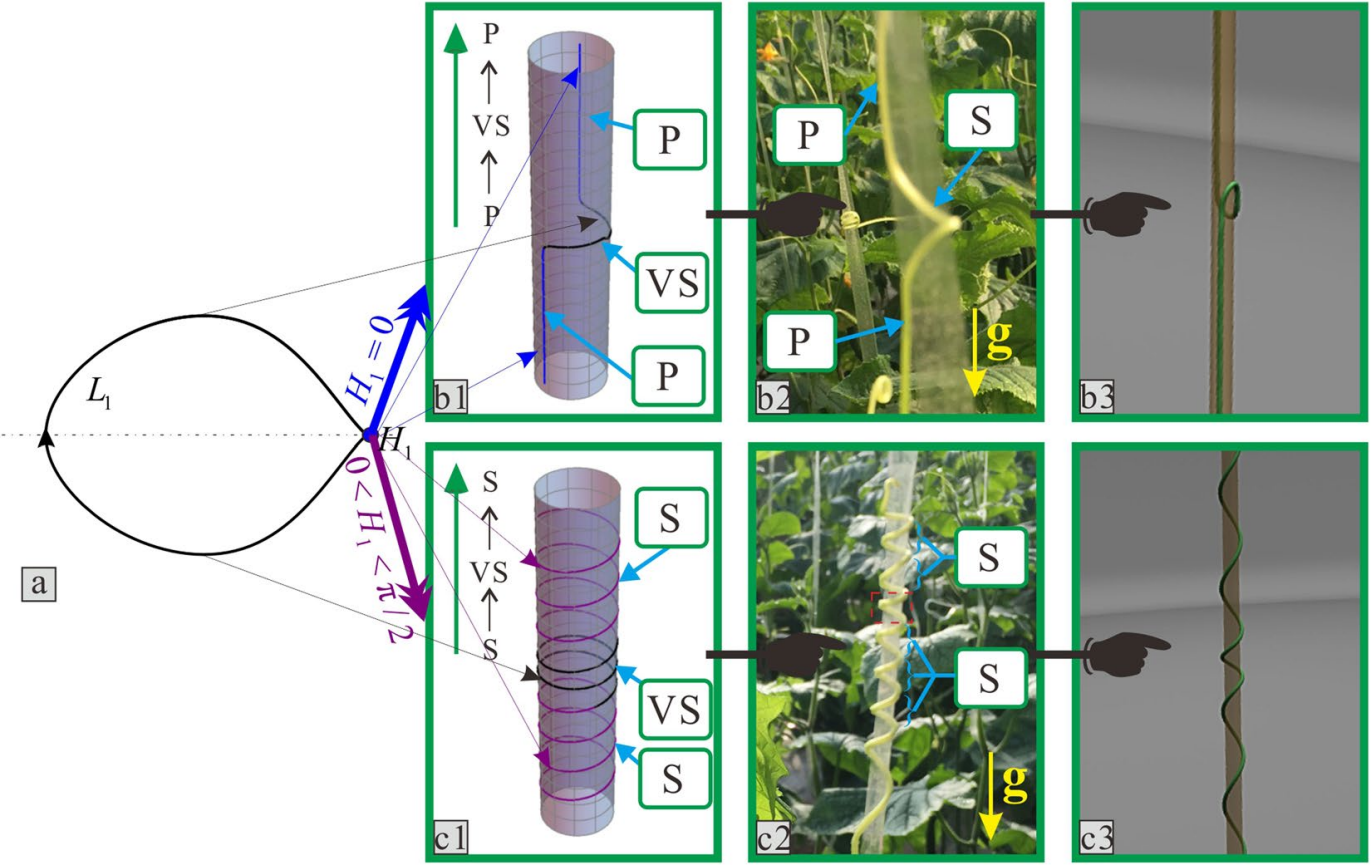
Relationships between the rod configuration and the growth state of the tendril corresponding to homoclinic orbits. (**a**) Homoclinic orbit. (**b**1) and (**c**1) Two types of theoretical configurations. (**b**2) and (**c**2) Two types of qualitative experimental results of tendril morphologies. (**b**3) and (**c**3) The three-dimensional diagrams corresponding to these two types. “VS” represents the spiral state with variable helix angle.

After this division, these combination configurations showed certain change rules. Cucumber tendrils with upward-right spiral growth in their natural state were used as example, and four types of morphology were qualitatively classified as shown in Figs 4(b2,c2) and 5(b2,c2). The letter “g” represents the direction of gravity. The letters **S** and **P** used previously are used to describe the combination configurations.

**Figure 5:**
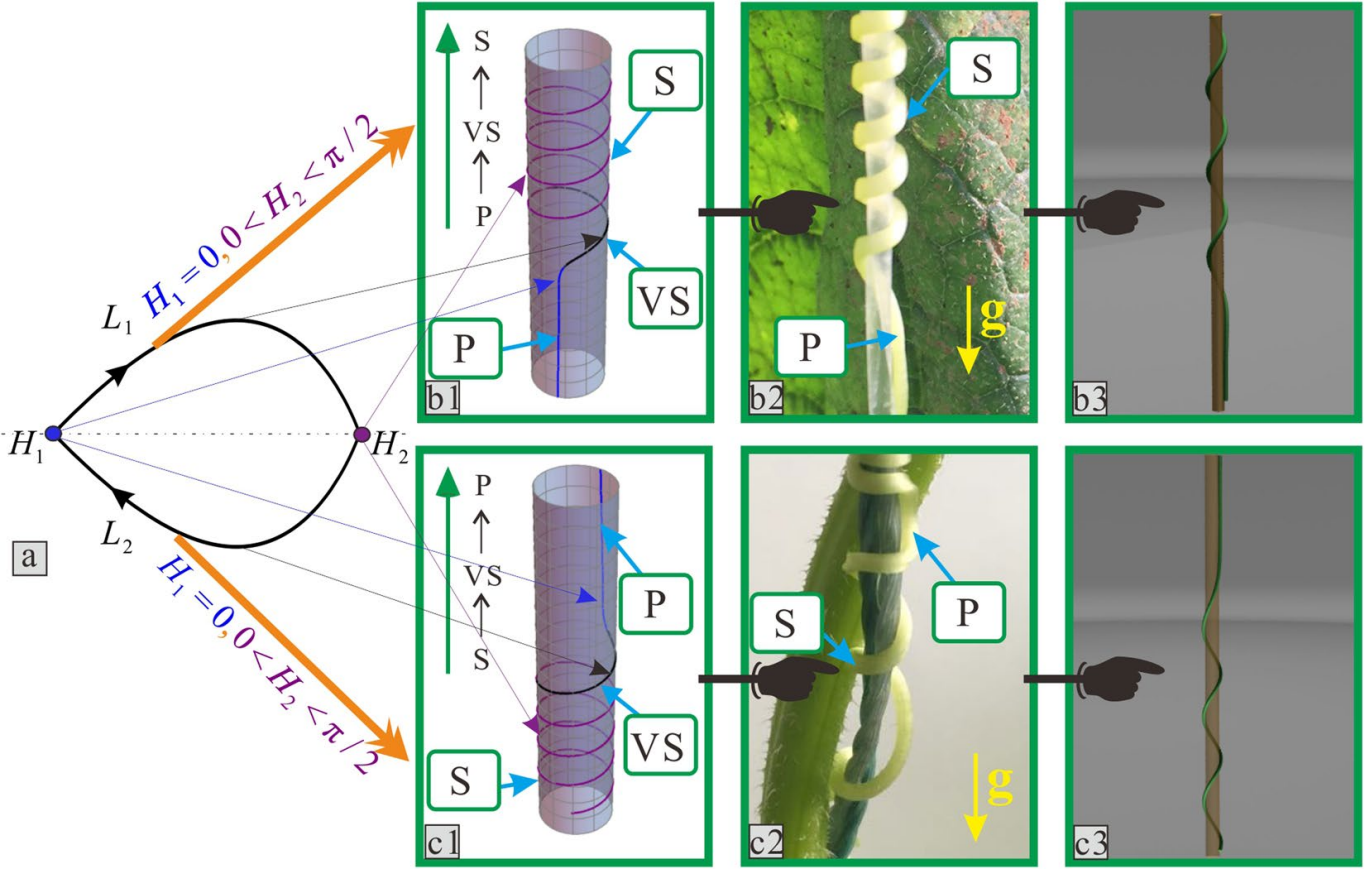
Relationships between the rod configuration and the growth state of tendril corresponding to heteroclinic orbits. (**a**) Heteroclinic orbits. (**b**1) and (**c**1) Two types of theoretical configurations. (**b**2) and (**c**2) Two types of qualitative experimental results of tendril morphologies. (**b**3) and (**c**3) The three-dimensional diagrams corresponding to these two types.

Case 1 is shown in Fig. 4(b2). The morphology of tendrils along the growth direction, which is the opposite direction of gravity, presents the combination form of the **P** → **S** → **P** in turn. The cases 2, 3, and 4 are shown in Figs 4(c2) and 5(b2,c2), respectively. The corresponding combination configurations of tendrils are shown as the **S** → **S**, the **P** → **S**, and the **S** → **P**, respectively.

## Rod Deformation and Characteristic Analysis

The force and deformation of tendrils are studied, as they deform from their initial form to their final form under load. In contrast to the previous growth model analysis, this section focuses on the two states. One is the initial state without deformation, which could be considered as a straight rod, and the other is the final state after deformation, and could be considered as a stable bent rod. The outcomes are discussed rather than the process.

### Equation of Deformation and Static Bifurcation

**Figure 6:**
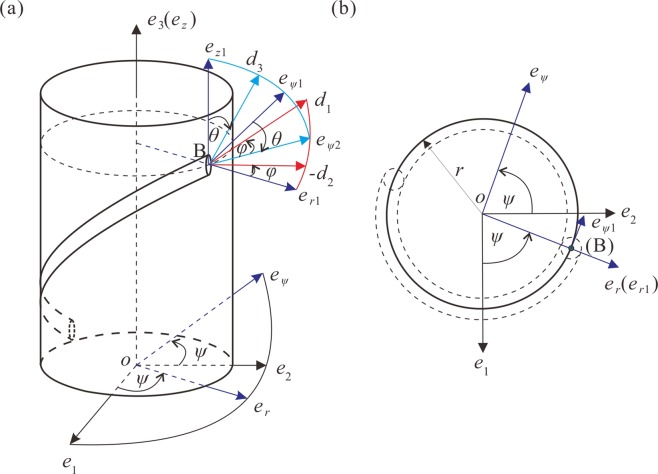
Coordinate positions and angles for constrained rod description. (**a)** Three-dimensional graphic. (**b**) Top view.

Assuming that a rod constrained by a cylinder is deformed under a specific applied load that are force *F* and force moment *M*, the coordinates of the system are presented in Fig. 6. The rod with a circular cross section smoothly contacts a cylinder, which provides the binding force along the normal direction.

The force of the rod is balanced. The vector function ***R*** is used to represent the position of the rod’s centre line in the coordinate system $\{{{\boldsymbol{e}}}_{1},\,{{\boldsymbol{e}}}_{2},\,{{\boldsymbol{e}}}_{3}\}$. As an element of the rod, through force analysis, the balance equations can be expressed via both the internal force ***n*** and moment ***m***:

15 $$\dot{{\boldsymbol{n}}}=0,$$

16 $$\dot{{\boldsymbol{m}}}={\boldsymbol{n}}\times \dot{{\boldsymbol{R}}},$$

where $(\dot{)}={\rm{d}}/{\rm{d}}s$, and *s* represents the arc length along the centre line of rod. The principal coordinate system of the centre line of the rod is denoted as $\{{{\boldsymbol{d}}}_{1},\,{{\boldsymbol{d}}}_{2},\,{{\boldsymbol{d}}}_{3}\}$, in which the vectors ***d***_1_ and ***d***_2_ respectively follow two directions of principal bending axes, and ***d***_3_ follows along the tangent direction of the rod. Thus,

17 $$\dot{{\boldsymbol{R}}}={{\boldsymbol{d}}}_{3}$$

The generalised strain ***u*** satisfies the following relationship:

18 $${\dot{{\boldsymbol{d}}}}_{i}={\boldsymbol{u}}\times {{\boldsymbol{d}}}_{i}\,(i=1,\,2,\,3).$$

The two components of ***u*** are curvatures and twist. The moment and strain satisfy the linear constitutive relation, which can be written as:

19 $${u}_{1}=\frac{1}{B}{\boldsymbol{m}}\cdot {{\boldsymbol{d}}}_{1},\,{u}_{2}=\frac{1}{B}{\boldsymbol{m}}\cdot {{\boldsymbol{d}}}_{2},\,{u}_{3}=\frac{1}{C}{\boldsymbol{m}}\cdot {{\boldsymbol{d}}}_{3},$$

where *B* and *C* represent the bending and torsional stiffness of the rod’s cross section, respectively.

For coordinate transformation, the Euler angle frame $\{\theta ,\psi ,\varphi \}$ was introduced and the determined cross section profiles are presented in Fig. 6. *θ* represents the angle between the rod’s centreline ***d***_3_ and the cylinder’s centreline ***e***_3_. *ψ* represents the circumferential angle the rod rotates around ***e***_3_. *ϕ* represents the twist angle of the rod at its cross section, which is the (***d***_1_, ***d***_2_) plane, and rotates around ***d***_3_. Considering the Cosserat director theory, the mathematical model of a rod constrained by a cylinder can be obtained by combining Eqs (15–18), and conducting a series of coordinate transformations^40,43,46,47^. The detailed equations are presented as Eq. (A1) in Supplemental Material 2.

Three boundary conditions apply to the system. The first implies that the axial moment should be balanced, since the rod is bound to a cylinder and cannot leave. The second implies that the Hamiltonian is a conserved quantity, because the rod is uniform. The third implies that the moment along the rod’s tangle direction should be balanced, because the rod’s cross section remains a circular plane after it twists around itself. These three conditions are constant and are denoted as *K*_1_, *K*_2_, and *K*_3_. These expressions in detail are written as Eqs (A3), (A4), and (A5) in Supplemental Material 2.

By combining these three boundary conditions, a reduced governing equation of deformation could be obtained via simplification:

20 $$\frac{1}{2}{\dot{\theta }}^{2}+V(\theta )=h,$$

where,

21 $$V(\theta )=\frac{\cos \,\theta }{f}+\frac{{K}_{1}\,\sin \,\theta }{\tilde{r}\,f}-\frac{{K}_{3}\,\sin \,\theta \,\cos \,\theta }{\tilde{r}}+\frac{{\cos }^{2}\theta }{\tilde{r}}-\frac{{\cos }^{4}\theta }{2{\tilde{r}}^{2}},$$

22 $$h={K}_{2}-\frac{1}{2}{K}_{3}^{2}(1+\nu )+\frac{1}{2{\tilde{r}}^{2}},$$

In Eq. (20), the non-dimensional parameters are as follows:

23 $$\tilde{r}=\frac{M\,r}{B},\,f=\frac{{M}^{2}}{B\,F},$$

These represent generalized distance and load, respectively. *r* represents the distance between the centrelines of the rod and the cylinder.

The governing Eq. (20) is very similar to a nonlinear dynamical equation, which contains complex nonlinear characteristics. Therefore, the qualitative existence of solutions to this governing equation can be discussed by applying the method for investigating changes in the system stability of nonlinear dynamics.

In such a stability analysis, a bifurcation diagram can be obtained according to the following condition:

24 $$\frac{\partial V(\theta )}{\partial \theta }=0.$$

To facilitate calculation, the boundary condition of one given point is:

25 $$\theta (0)=0,\,\dot{\theta }(0)=0.$$

In combination with the experimental data presented in the previous section, the two new dimensional key functions are chosen as follows:

26 $$x=\frac{\pi \,EF{r}^{2}}{64B},$$

27 $$y=\frac{\pi \,EMr}{64\,B},$$

**Figure 7:**
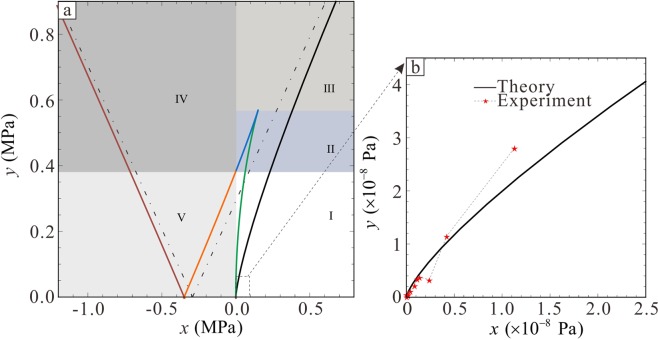
Experimental data and theoretical prediction of growth experiment. (**a**) Theoretical bifurcation sets of *x*-*y* domains when the potential energy of one singular point is identical to the potential energy of the given point. (**b**) Comparison of theoretical and experimental results when tendrils are winding a knot.

When a solution of the governing Eq. (20) exists, the relationship between $\mathop{r}\limits^{\sim }$ and *f* can be calculated and transformed into the relationship between functions (26) and (27). This is plotted via static bifurcation calculation, as shown in Fig. 7.

In Fig. 7(a), five colour curves correspond to five critical cases. According to the number of critical cases, the entire diagram could be divided into five regions. Region III and V have one critical case, region I and III have two and region II has three. Three sets of critical cases exist in region III, which are represented by the black, green, and blue curves in Fig. 7(a). Two sets exist in region V, which are represented by the orange and brown curves in Fig. 7(a). Therefore, regions III and V are used as examples without loss of generality.

### Homoclinic and Heteroclinic Orbits in Rod Deformations

The static and dynamic stability of solutions in governing Eq. (20) are discussed by analysing the potential energy curves and phase diagrams from a nonlinear dynamics point of view. Here, the focus is on the angle *θ*, not on the arc length *s*.

The value of the boundary conditions can be assumed as the following^40,43,46,47^:

28 $${K}_{1}=f,\,{K}_{2}=\mathrm{Free},\,{K}_{3}=1.$$

**Figure 8:**
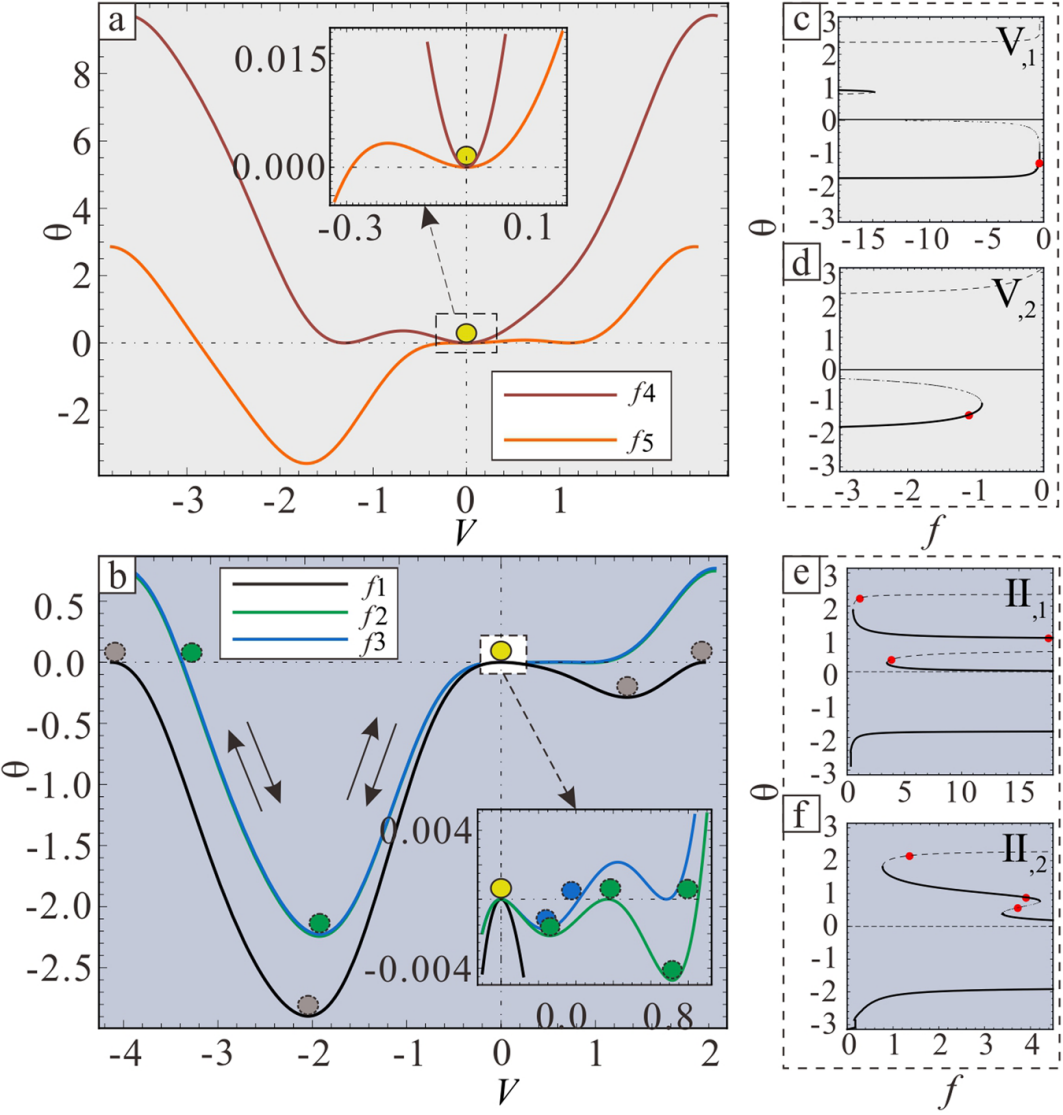
Stability analysis in $\tilde{r}\in {\rm{V}}$ and $\tilde{r}\in {\rm{II}}$. (**a**,**b**) Potential energy curves in region IV and II, respectively. (**c**) and (d) Two types of *f* − *θ* domains in region IV; region IV_1_ and IV_2_ are $\tilde{r}=0.68$ and $\tilde{r}=1.5$, respectively. (**e**,**f**) Two types of *f* − *θ* domains in region II; region II_1_ and II_2_ are $\tilde{r}=0.68$ and $\tilde{r}=0.9$, respectively. The solid line indicates a stable solution, while the dashed line indicates an unstable solution. The red dot indicates the critical value.

Then, the potential energy curves can be plotted as Eq. (21) and the zero potential energy is at the origin, as shown the diagrams *θ*–*V(θ)* in Fig. 8(a,b).

In region III, the given point is the unstable solution where the root of zero corresponds to the dashed line in Fig. 8(e,f) with regard to the stability of the static solution. If a stable dynamic solution exists, the system would have a stable solution. From an energy point of view, a ball that maintains its original energy can move globally along its potential energy curve as far as it reaches the same level of original energy as shown in Fig. 8(b); this represents the global motion. Then, the global critical orbits with dynamical stability, which can be called critical solutions or HAHOs, appear in these three critical cases. Therefore, from analytical discussions to actual tendrils, this also indicates that the tendril will not remain in the configuration of the unstable solution. This demonstrates a specific stable buckling behaviour corresponding to HAHOs.

In region V, the given point is the stable solution where the root of zero corresponds to the solid line in Fig. 8(c,d). Furthermore, a ball is located and initially only stays at the given point, because the ball cannot escape from both ends of the barrier, as shown in Fig. 8(a). Then, a critical orbit cannot be found through the given points in these two critical cases. Therefore, this indicates that the tendril can only maintain the morphology corresponding to the given point.

**Figure 9:**
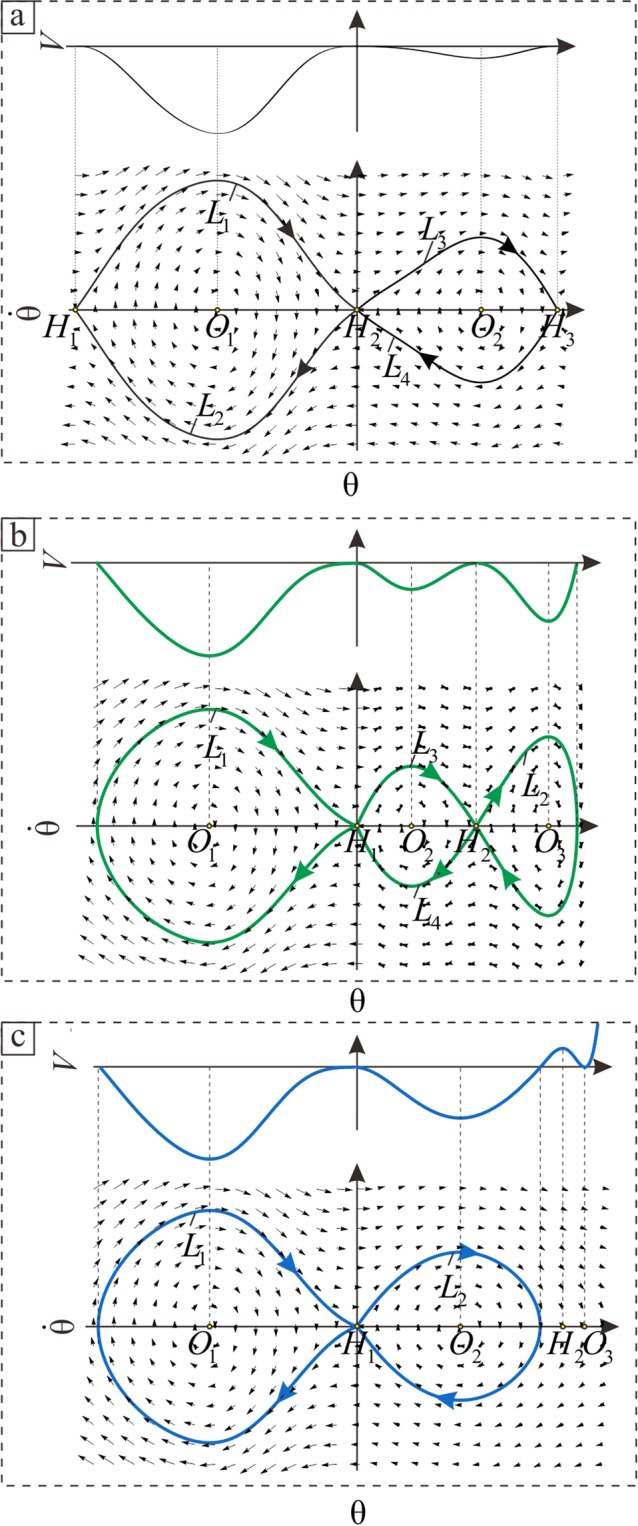
The structure diagrams of potential energy curves and phase diagrams of critical orbits. (**a**) Type one. (**b**) Type two. (**c**) Type three.

The phase diagrams $\theta -\dot{\theta }$ can be drawn by Eqs (20) and (25). The relationship between potential energy curves and phase diagrams are shown in Fig. 9. This cuts the potential curve horizontally from zero. Three typical structure diagrams of phase diagrams in Fig. 9, which correspond to the black, green, and blue curves in Fig. 8(b), represent the HAHOs in three critical cases that belong to region III. Figure 9 shows four homoclinic orbits that include the *L*_1_ and *L*_2_ in Fig. 9(b,c), respectively; furthermore, there are six heteroclinic orbits that include the *L*_1_, *L*_2_, *L*_3_, and *L*_4_ in Fig. 9(a) and *L*_3_ and *L*_4_ in Fig. 9(b).

To not break away from the original tendril morphology problem, namely the deformation of the rod, the angle *θ* discussed above needs to be returned to the original physical Eq. (A1). Therefore, the configuration of the rod can be plotted by the following coordinate transformation:

29 $$X=r\,\cos \,\psi ,\,Y=r\,\sin \,\psi ,\,\dot{Z}=\,\cos \,\theta .$$

In this way, the configurations of solutions in the governing Eq. (20) can be discussed. Structure diagrams of rod configurations are utilized to illustrate the distribution relationships among the saddle point, centre point, homoclinic orbit, and heteroclinic orbit in Figs 4 and 5. Four types of rod deformation results exist when the solutions in the governing Eq. (20) are on different saddle points and HAHOs.

The configurations of rods corresponding to the four homoclinic orbits in Fig. 9 were divided into two types. One is drawn as Fig. 4(b1), and represents the *L*_1_ in Fig. 9(b) and *L*_1_ and *L*_2_ in Fig. 9(c). The other is drawn as Fig. 4(c1), and represents the *L*_2_ in Fig. 9(b). There are two types with heteroclinic orbits in Fig. 9. The first is the *L*_3_ in Fig. 9(b), as shown in Fig. 5(b1). The second is the *L*_4_ in Fig. 9(b), as shown in Fig. 5(c1). Here, the type one critical orbits are not discussed in detail. This is because, via analysis, its characteristics are similar to a previously presented conclusion^46^. This is the knot phenomenon. Thus, all solutions, other than type one, are qualitatively summarized.

## Numerical Results and Discussion

The tendril morphologies obtained in actual growth and the configurations of the theoretically predicted rod are presented together for comparison to illustrate the existence of HAHOs. The tendril morphology and HAHOs are indeed related from the results of theoretical and experimental analysis.

Firstly, the data from the growth experiments are compared with theoretical results. The data in Table 1 and the Eqs (13) and (14) are used to draw the experimental data graph corresponding to Eqs (26) and (27), and then, these two functions can be rewritten into the form related to the experimental data as follows:

30 $$x=\frac{F}{{d}^{4}}{(\frac{D-d}{2})}^{2},$$

31 $$y=\frac{M}{{d}^{4}}{(\frac{D-d}{2})}^{2}.$$

Figure 7(b) shows a comparison between the theoretical results and the experimental results when the knotting phenomenon occurs. The red pentagram marks represent the experimental results, and the black solid lines represent the theoretical results, showing a locally enlarged view of the black curves in Fig. 7(a). The experimental data and the theoretical prediction data show similar trends, when the actual growth tendrils show the knot phenomenon; this corroborates the validity of the analytical framework.

Secondly, the data from the morphological experiments are compared with the theoretically predicted results. The rod configurations corresponding to HAHOs are compared to the actual morphologies of the tendril to explain the potential nonlinear mechanism of tendril growth.

In Figs 4 and 5, the value of the saddle points determines the spiral angle located at both ends of the rod, which are represented by the same colour. The size of HAHOs, which represents the stage from one saddle point to another, determines the azimuth angle of the black colour arc, which is the middle part of the rod, represented by the black curve in Figs 4(b1,c1) and 5(b1,c1). At this stage, the spiral angle of the rod (constrained by a cylinder) is variable, and was marked with the letters “**VS**”.

When the point is located at the saddle point (0, 0), which is represented by the blue point *H*_1_ in Figs 4(a) and 5(a), the spiral angle of the corresponding point on the configurations of the rod is 0°. As in the blue curves in Figs 4(b1) and 5(b1,c1), one end of the rod displays a similarly straight shape. For the tendril, one end of the tendril morphology displays a parallel state along the centreline of the support, which is marked with the letter **P** in Figs 4(b2), 5(b2,c2). If the point is not located at this saddle point, both the rod and tendril are all at the spiral state.

When the homoclinic orbit occurs, the helix angle at both ends of the rod retains the same constant value *H*_1_; the helix angle at the middle part of the rod is variable. Fig. 4(b1) is similar to the case 1 mentioned in the morphological experiments as shown in Fig. 4(b2). If the saddle point *H*_1_ = 0, firstly, the point originates at this saddle point, and the tendril initially climbs upward along the support in a parallel state, which is **P**. Then, when the point passes through the homoclinic orbit *L*_1_, the tendril continuously climbs and starts to rotate around the support at a variable helix angle and in a spiral manner, which is **VS**. Finally, when the point returns to this saddle point, the tendril movement returns to **P**. According to this growth pattern, case 1 is called climbing-upward. Fig. 4(c1) is similar to case 2 in Fig. 4(c2). If the saddle point $0 < {H}_{1} < \pi /2$, the motion of the point in orbit *L*_1_ is identical to case 1, but the tendril grows differently. Firstly, the tendril climbs upwards along the support in a spiral state with an initially constant helix angle, which is **S**. Subsequently, the tendril grows in the manner of **VS**. Finally, the tendril movement returns to **S**. Therefore, it is called fixing-upward.

When the heteroclinic orbit occurred, the helix angle at both ends of the rod that spirals around the cylinder always retains its corresponding constant values *H*_1_ and *H*_2_, and that at the middle part of the rod is variable. Fig. 5(b1) is similar to case 3 in Fig. 5(b2). If the point moves along the heteroclinic orbit *L*_1_, while the saddle points are *H*_1_ = 0 and $0 < {H}_{2} < \pi /2$, firstly, the point originates at the saddle point *H*_1_ and the tendril climbs upward and initially grows in the manner of **P**. Then, when the point passes the orbit *L*_1_, the tendril continuously climbs upward and grows in the manner of **VS**. Finally, when the point arrives at the saddle point *H*_2_, the tendril grows in the manner of **S**. Therefore, this climbing mode is called climbing-fixing-upward. The Fig. 5(c1) is similar to case 4 of Fig. 5(c2). If the point moves along the heteroclinic orbit *L*_2_, the point originates at the saddle point *H*_2_ and arrives at the saddle point *H*_1_. The tendril grows upward as a reverse version of case 3. The tendril grows following the manner of **S** to **VS** and finally returns to **P**. Therefore, this is called fixing-climbing-upward.

The morphology of the tendril obtained in the actual growth is similar to the rod’s theoretically predicted configuration, which corroborates the validity of the analytical framework again. Upward-right spiral growth is used as example; however, the application is not limited to this type of growth.

In addition, to provide a more accessible understanding of the processes above, the growth processes of tendrils are obtained by drawing their three-dimensional coordinate data, as shown in Figs 4(b3,c3) and 5(b3,c3). The animation simulations of tendril winding to support growth are shown in Supplemental material 3–6. The four cases of these growth processes of a tendril are obtained by Unigraphics NX and Adobe Premiere Pro. The three-dimensional coordinate data of the rods obtained via theoretical results in the above-mentioned four groups are derived with the use of actual tendrils physical parameters.

## Conclusion

The analytic results indicate that the morphology of climbing tendrils indeed correlates with a similarly complex nonlinear behaviour. HAHOs phenomena were found in the growth of climbing cucumber tendrils. The chirality and parallel growth combination configurations in tendrils can be reasonably and effectively explained from the point of view of HAHOs.

Cucumber tendrils are biological materials that exhibit properties of plastic materials during their tensile state. The average elastic modulus of a tendril is about 12 MPa, when its average diameter is close to 1.175 mm. Tendril growth is sufficiently slow that the deformation of a rod constrained by a cylinder can be used to model the growth of a tendril climbing a support.

The validity of the utilized analytical framework has been demonstrated in two ways. When the actual growth of tendrils shows the knot phenomenon, theoretic curves achieve good coherence to experimental data. When tendrils show combination configurations with upward-right spiral growth, their morphologies summarized via both experiment and theory are qualitatively consistent.

The four types of growth morphologies can be called climbing-upward, fixing-upward, climbing-fixing-upward, and fixing-climbing-upward. According to the entire growth pattern, five critical theoretically predicted cases can be classified. The orange and brown curves can be called the contact. The black and blue curves can be called knot and climbing-upward, respectively. The green curves include four growth morphologies as described above.

In future, the quantitative difference could be improved by comparing the tendril growth process of different regions to further determine the dimensionless coefficient in the model equations. The obtained analytic results provide a powerful theoretical support for climbing problems; therefore, these results could also be utilized to address other research directions, such as the drill pipe control of an offshore drilling platform, the macromolecular entanglement structure in pharmaceutical research, and the growth control of novel materials.

In summary, the growth of plants is not only an adaptation to their environment, but also potentially contains many scientifically significant phenomena. Nonlinear problems are ubiquitous and HAHOs are not only theoretical concepts.

## Supplementary information

Supplemental material 1

Supplemental material 2

Supplemental material 3

Supplemental material 4

Supplemental material 5

Supplemental material 6
