# Supplementary material for: Homoclinic and Heteroclinic Orbits in Climbing Cucumber Tendrils
Source: Sci Rep. 2019 Mar 25;9:5051. doi: 10.1038/s41598-019-41487-5 (PMC6433869; doi:10.1038/s41598-019-41487-5)
Supplement: Supplementary file 2 — Supplemental material 2 [file 41598_2019_41487_MOESM2_ESM.docx]

**SUPPLEMENTARY MATERIAL**

**Homoclinic and Heteroclinic Orbits in Climbing Cucumber Tendrils**

Jingjing Feng ^1, 2^, ^3, *^ , Wei Zhang ^2,*^ , Cheng Liu ^1, 3^, Ming Guo ^4^, Chunqiu Zhang ^1, 3^

^1^Tianjin Key Laboratory for Advanced Mechatronic System Design and Intelligent Control, School of Mechanical Engineering, Tianjin University of Technology, Tianjin 300384, China;

^2^Beijing Key Laboratory on Nonlinear Vibrations and Strength of Mechanical Structures, Beijing University of Technology, Beijing 100124, China;

^3^National Demonstration Center for Experimental Mechanical and Electrical Engineering Education, Tianjin University of Technology, Tianjin 300384, China;

^4^Tianjin KunLun Decoration Engineering Company, Tianjin 300191, China.

^*^Correspondence and requests for materials should be addressed to J.-J.F. (jjfeng@tju.edu.cn) or W.Z. (wzhang@bjut.edu.cn)

**The physical and mathematical model**

The rod with a circular cross section smoothly contacts the cylinder which could provide the binding force along the normal direction. The $\psi$, $\theta$, and represent the Euler angles, as presented in Fig.4. According to the modelling of the Cosserat director theory and though coordinate transformations ^40, 43, 47^, the mathematical model of an elastic rod constrained by a cylinder can be written as follows:

where the dimensionless parameters are as follows:

$$\begin{matrix} x_{1}=\frac{N_{r}}{T},x_{2}=\frac{N_{\psi}}{T},x_{3}=\frac{N_{z}}{T},x_{4}=\frac{M_{r}}{M},x_{5}=\frac{M_{\psi}}{M},x_{6}=\frac{M_{z}}{M}, \\ t=\frac{M}{B}s,\tilde{H}=\frac{H}{T},f=\frac{M^{2}}{B_{2} T},\nu=\frac{B}{C}-1,\tilde{r}=\frac{M}{B}r. \end{matrix}$$

$\nu$ represents Poisson's ratio of a rod. *H* and *N_i_* (*i*=1,2,3) are coefficients introduced in the derivation. According to the detailed derivation in previous studies ^40, 43, 46, 47^, the $x_{1}=\tilde{H}$ and $x_{3}=1$ could be gained.

The three boundary conditions could be written as follows,

$K_{1}=x_{2}\tilde{r}+f x_{6}=\text{Constant}$,

$K_{3}=x_{5}\sin\theta+x_{6}\cos\theta=\text{Constant}$.

And then the original system *equation reference goes here* could be reduced to the following governing equation ,

where,

$$\left\{\begin{matrix} V(\theta)=\frac{\cos\theta}{f}+\frac{K_{1}\sin\theta}{\tilde{r} f}-\frac{K_{3}\sin\theta\cos\theta}{\tilde{r}}+\frac{\cos^{2}\theta}{\tilde{r}^{2}}-\frac{\cos^{4}\theta}{2\tilde{r}^{2}}, \\ h=K_{2}-\frac{1}{2}K_{3}^{2}(1+\nu)+\frac{1}{2\tilde{r}^{2}}. \end{matrix}\right.$$
