# Supplementary figures and images for: Homoclinic and Heteroclinic Orbits in Climbing Cucumber Tendrils
Source: Sci Rep. 2019 Mar 25;9:5051. doi: 10.1038/s41598-019-41487-5 (PMC6433869; doi:10.1038/s41598-019-41487-5)

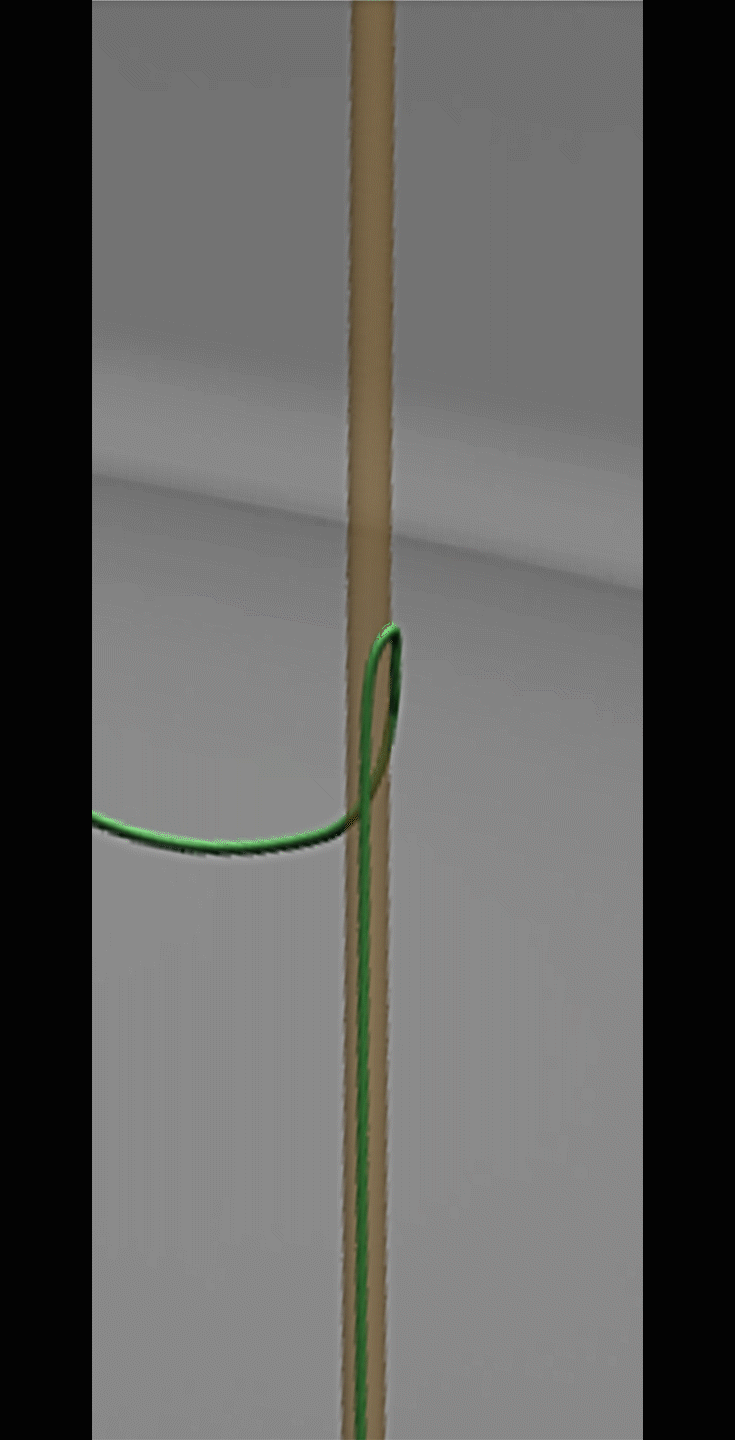

Supplement: Supplementary file 3 — Supplemental material 3 [file 41598_2019_41487_MOESM3_ESM.gif]

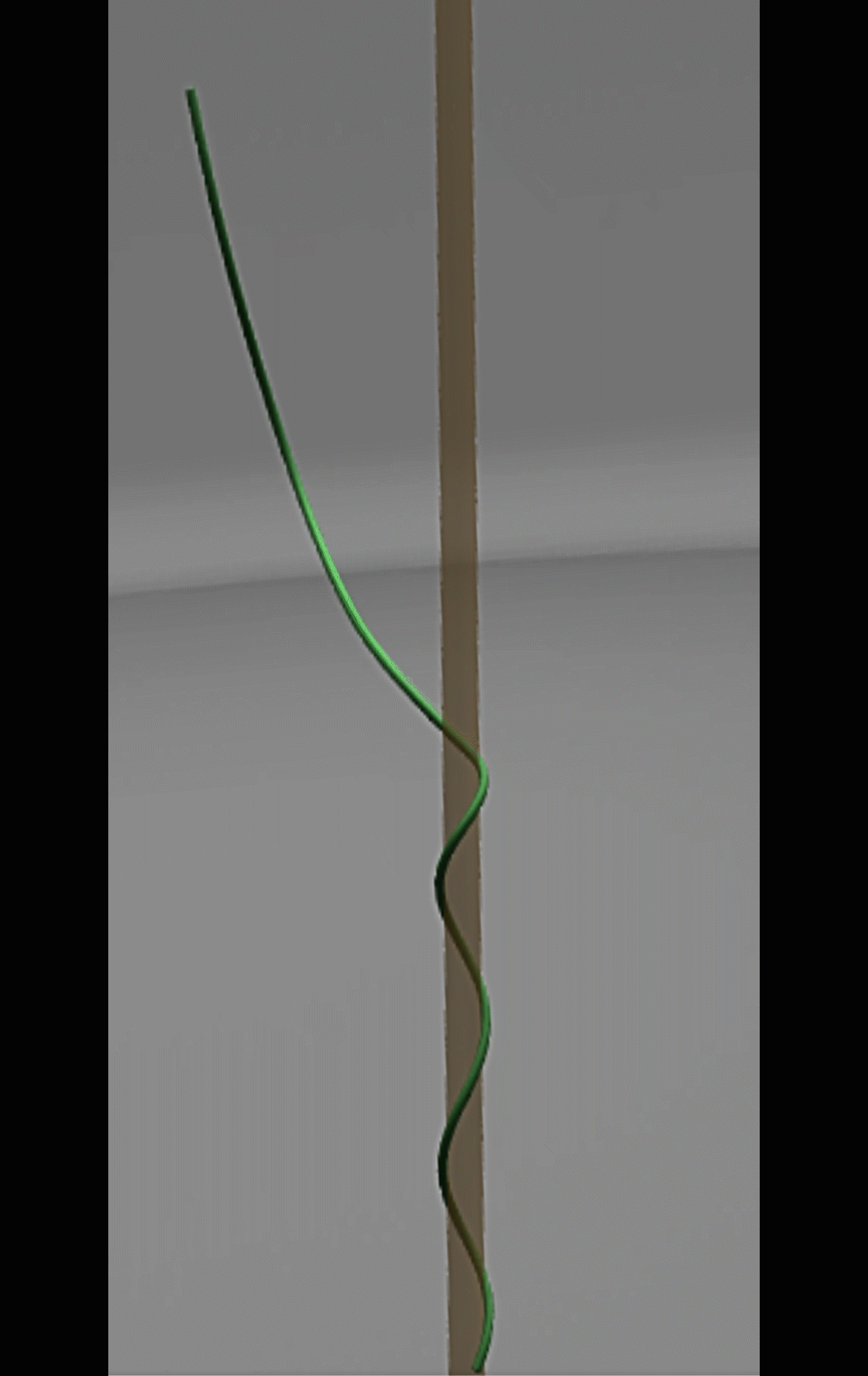

Supplement: Supplementary file 4 — Supplemental material 4 [file 41598_2019_41487_MOESM4_ESM.gif]

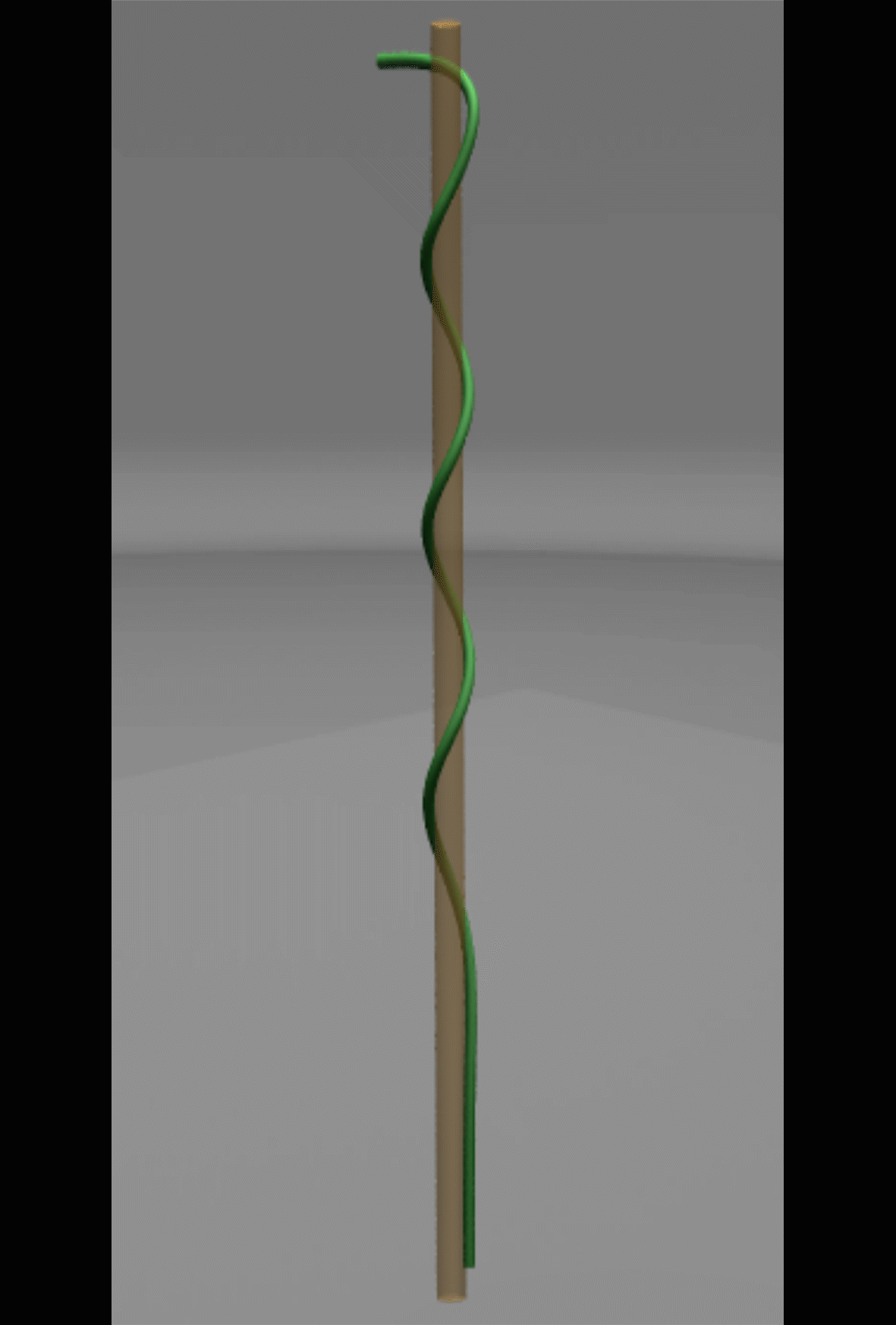

Supplement: Supplementary file 5 — Supplemental material 5 [file 41598_2019_41487_MOESM5_ESM.gif]

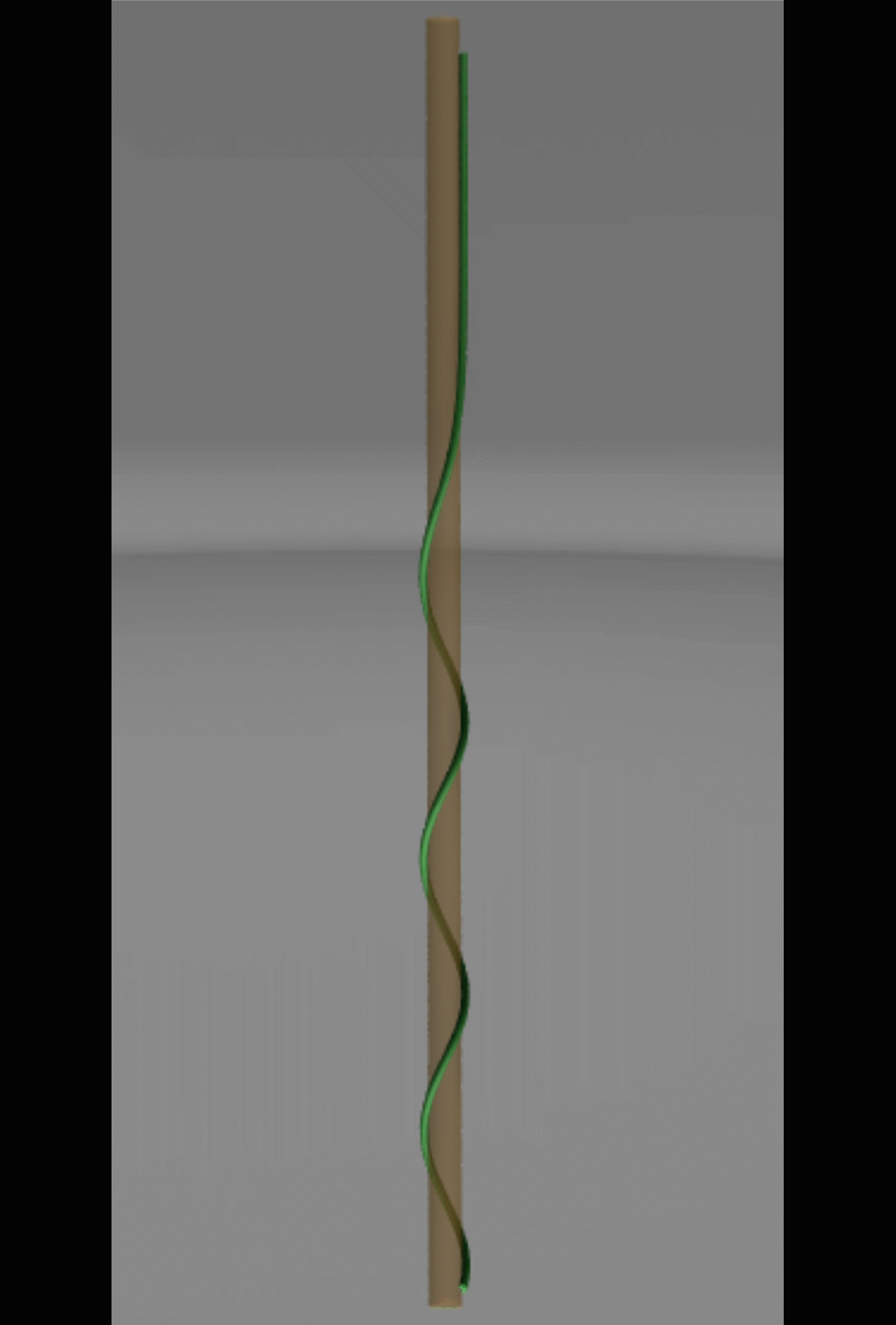

Supplement: Supplementary file 6 — Supplemental material 6 [file 41598_2019_41487_MOESM6_ESM.gif]
